# Changes in Daily Steps and Body Mass Index and Waist to Height Ratio during Four Year Follow-Up in Adults: Cardiovascular Risk in Young Finns Study

**DOI:** 10.3390/ijerph14091015

**Published:** 2017-09-05

**Authors:** Kasper Salin, Mirja Hirvensalo, Costan G. Magnussen, Risto Telama, Nina Hutri-Kähönen, Jorma Viikari, Olli Raitakari, Tuija Tammelin

**Affiliations:** 1 Faculty of Sport & Heath Sciences, University of Jyväskylä, 40014 Jyväskylän, Finland; mirja.hirvensalo@jyu.fi (M.H.); risto.r.telama@jyu.fi (R.T.); 2 Research Centre of Applied and Preventive Cardiovascular Medicine and Departments of Clinical Physiology and Internal Medicine, University of Turku and Turku University Central Hospital, 20500 Turku, Finland; costan.magnussen@utas.edu.au (C.G.M.); jorma.viikari@utu.fi (J.V.); olli.raitakari@utu.fi (O.R.); 3 Menzies Institute for Medical Research Hobart, University of Tasmania, Hobart, TAS 7000, Australia; 4 Department of Pediatrics, University of Tampere and Tampere University Hospital, 33100 Tampere, Finland; nina.hutri-kahonen@uta.fi; 5 LIKES Research Centre for Physical Activity and Health, 40014 Jyväskylä, Finland; tuija.tammelin@likes.fi

**Keywords:** physical activity, pedometer, adults, follow-up, body mass index, waist-to-height ratio

## Abstract

*Aims:* Over the study years, there was a significant increase in body mass index (BMI) and waist-to-height ratio (WtHR) in middle aged Finnish adults. *Methods:* Data were obtained from 1033 Finnish adults from the Cardiovascular Risk in Young Finns Study in 2007 and 2011. Cohort study participants wore an Omron Walking Style One (HJ-152R-E) pedometer for five days and were grouped into those who increased, maintained and decreased their steps between 2007 and 2011. Paired samples *t*-test was used to compare body mass index (BMI) and waist-to-height ratio (WtHR) change values between the change groups in study years. *Results*: Among study population BMI and WtHR increase between study years was statistically significant (*p* < 0.001). Only those, who increased their total steps for at least 2000 steps, maintained their BMI in the same level, while people who decreased or maintained their total steps in the same level, BMI and WtHR increased during four years follow-up. *Conclusions:* This data suggests that increasing steps in middle age is associated with maintaining BMI at the same level.

## 1. Background

In recent decades, physical inactivity has emerged as a major health problem. Recent data show physical inactivity to be the fourth leading cause of death worldwide [1], with 6–10% of all deaths from non-communicable diseases worldwide being attributable to physical inactivity [2,3]. Physical inactivity is also associated with obesity and other major risk factors for chronic disease [2]. Although leisure time physical activity (PA) has increased, occupational PA has decreased in high income and rapidly developing countries with current estimates suggesting that 31% of the world’s population is not meeting PA recommendations [4]. During the 20 years follow-up (1975–2014) of 19.2 million participants, BMI increased from 21.7 to 24.2 among men and from 22.1 to 24.4 among women [5]. Overall, the worldwide proportion of adults with a BMI of 25 kg/m^2^ or greater have increased from 28.8% to 36.9% in men and from 29.8% to 38.0% in women between the years 1980 and 2013 [6].

Based on self-reported questionnaires, 48.6% of adults in Finland do not meet the recommendations for health enhancing physical activity (HEPA) [7]. However, self-reported questionnaires of PA have several limitations when compared with objectively measured PA [8]. Recent data have emerged from studies with objective measurements of PA [9,10,11,12] that suggest a decline of total PA and an increase of sedentary time among adults in economically developed countries. However, there are signs of gender and age differences between countries. In some countries, the decline in daily steps have been observed only among men [9], women [10], or in certain age groups [9]. 

Obesity is often considered to be a result of either excessive food intake or insufficient physical activity [13]. High sedentary time is related to higher body weight and increased risk of obesity [14]. In addition, low PA has been found to be a risk factor for higher prevavelnce of adverse cardiometabolic health indicators [15]. Recent studies using objective measurements of PA have found that short term changes in daily steps do not influence body mass index (BMI) [16]. Similarly, even though individuals have increased their PA, the increase has not been enough to prevent weight gain [17]. Likewise among obese individuals, the trend of daily steps is stable or even declining while among normal and overweight people trend of daily steps is increasing among the adult population in the USA [18]. In recent studies, it has been stated that alongside BMI, other measures should be used to measure central obesity, like waist circumference, and waist-to-hip ratio [19] and waist-to-height ratio (WtHR) [20]. No research has examined the associations between PA change over a longer time period using objective measurements with BMI, and WtHR in population-based samples. Therefore, the aim of this study is to evaluate changes in PA during a four years follow-up period among Finnish adults and explore the associations between changes in daily steps with changes of BMI and WtHR.

## 2. Methods

### 2.1. Study Design and Participants

Data was obtained from the ongoing cohort study, Cardiovascular Risk Young Finns Study (YFS) that began in 1980 [21]. The population of this study consisted of women and men aged 30, 33, 36, 39, 42 and 45 years who participated in the 2007 and 2011 follow-ups. In total, there were 2204 participants who attended clinics in 2007 of which 1874 (85.0%) completed the pedometer study. In 2011, 2005 (55.7% of the baseline participants) participated in study clinics, of whom 1525 completed the pedometer study (76.1%). Participants who had at least 5 recorded days and at least 8 h per day of pedometer wear time in both 2007 and 2011 were included. Therefore, step data from 1033 participants was used. From these participants, 1033 also had weight and height data available. The study participants gave written informed consent in accordance with the Helsinki Declaration and the study protocol was reviewed and approved by the ethics committee of the participating universities (Decision number 533/2006).

### 2.2. Anthropometric Measures

Height, weight, and waist circumference were measured by educated and experienced measurers (e.g., nurses). Body mass index (BMI) was calculated as weight (kg)/height (m)^2^. Waist-to-height ratio (WtHR) was calculated as waist (cm)/height (cm). 

### 2.3. Measurement of Physical Activity

Participants were issued an Omron Walking Style One (HJ-152R-E) pedometer and were instructed to attach the pedometer during waking hours on their waistband or belt in the same position for seven consecutive days and to maintain a pedometer log.

Based on questionnaire data, the level of self-rated PA did not differ significantly between those who participated and who did not participate in the pedometer study in 2007. The study participants gave written informed consent and the study protocol was reviewed and approved by the ethics committee of the participating universities.

Pedometer logs were used to record the time of pedometer removal and at the end of the day to record the steps taken on the display. Participants were asked to continue with their typical activities and to remove the pedometer only while bathing or swimming. Participants could report comments and problems about their pedometer use in the pedometer log and could contact the study personnel. On the 8th day, participants were instructed to send their pedometer log and the pedometer to the study centers using a padded mailbag in a self-addressed, stamped envelope that was provided to all participants.

The Omron pedometer collects aerobic steps and minutes in addition to total steps. Aerobic steps are those taken during activities that last for at least ten minutes without interruption at a pace of 60+ steps per minute. We compared Omron Walking Style One pedometers with the steps measured by ActiGraph accelerometers (GT1M) in a subsample of 45 participants for 6 to 7 successive days (total of 304 days). The Spearman’s rank correlation coefficient was 0.966 (*p* < 0.001) [22].

All those who had at least five recorded days with a wear time at least 8 h, were included in all analyses. Sickness or injury status, exceptional step counts reported as an untypical day, or problems with pedometer use, were considered and compensated by the mean of other days. A daily wear time was imputed if the participant had reported at least two days wear time for eight hours. Participants reported several reasons for daily nonparticipation or interruption to pedometer wear. The main reasons in 2007 were lost (*n* = 52) or broken pedometer (*n* = 23), illness (*n* = 30), or other reasons such as untypical day (*n* = 22). The remainder of the participants (*n* = 203) chose not to participate in the pedometer study or did not send pedometer information back to the research center. In 2011, main reasons for nonparticipation were; lost device (*n* = 1), illness (*n* = 105, and other reasons such as untypical day (*n* = 31). The remainder of the participants (*n* = 388) chose not to participate in the pedometer study or did not send pedometer information back to the research center. The final sample size was 1518 imputed and corrected steps in 2007 and 1359 in 2011. Together, there were 1033 participants who had proper data from both years. There was no seasonal variation in total steps between summer and winter months neither in 2007 (*p* = 0.677) nor 2011 (*p* = 0.059) and wear time was considered in the analyses. 

Total steps and aerobic steps were used as continuous variables in descriptive analysis and in correlation analyses but as categorical variables when analyzing upward and downward proportions of change from 2007 to 2011. Changes in daily steps between the follow-up (2007 & 2011) were categorized to two main categories (increasers and decreasers) and these were divided to three more subcategories depending on the amount of increase/decrease (>2000/1000–1999/0–999 steps/day). In further analysis, these groups were merged to three categories increasers (>2000 steps/day or more), decreasers (>2000 steps/day or more) and maintainers (0–1999 steps/day increase or decrease). The same categories were also used in the comparison of BMI change among participants. Aerobic steps were classified in three categories according to change in aerobic steps/day between 2007 and 2011 (1) Maintainers (increase or decrease <1000 steps/day), (2) Decreasers (decline > 1000 steps/day), and (3) Increasers (increase >1000 steps/day) (Table 1).

### 2.4. Statistical Analysis

Participant descriptives for 2007 and 2011 are shown as mean ± SD for normally distributed variables and median (25th, 75th percentile) for total and aerobic steps per day owing to right-skewed distributions. Correlation between step changes and BMI changes (and WtHR change) were calculated with Pearson’s correlation to find out linear association between PA and BMI and WtHR change. Pearson’s correlation was used to find whether there are associations between BMI and WtHR changes and step changes. Proportion of participants in increasers’, maintainers’, and decreasers PA groups (meeting the three categories described above) are displayed as proportions for males, females and the total sample. Paired samples *t*-test was used to compare BMI values among those who decreased, increased or maintained their steps between 2007 and 2011. All statistical analyses were performed using SPSS version 22.0 (IBM, New York, NY, USA). Significance level was set to 0.05.

## 3. Results

Table 1 shows the proportions of participants across the change in step categories. 25.3% of participants (women = 24.8%, men 25.8%) increased and 18.7% (women = 16.0%, men = 21.5%) decreased their activity by at least 2000 total steps between 2007 and 2011. There was no difference in the proportion of men and women between the increase and decrease change groups (*p* = 0.726). Totally, 47.9% of the participants (women = 44.8%, men = 51.2%) did not change aerobic steps per day (±<1000 steps) while about one fourth decreased or increased their aerobic steps. Among women there were more changes in aerobic steps than among men (*p* < 0.001). A higher proportion of women either decreased (30.1%) or increased (25.1%) their aerobic step counts compared with men (decreased, 24.6%; increased, 24.2%).

Table 2 shows the characteristics of the 1033 participants included in the final sample. In 2007, participants averaged 7713 total steps/day and 1999 aerobic steps/day. In 2011, participants averaged 8112 total steps/day and 1986 aerobic steps/day. Overall, 56.5% of participants had more total steps/day in 2011 than 2007 and 51.5% had more aerobic steps/day in 2011 than 2007 (*p* < 0.001). In 2007, 19.0% of adults were active (more than 10,000 steps daily), while in 2011 the percentage was 24.8, (*p* < 0.001). In 2007, 48.9% of participants were inactive (<5000 steps per day) compared with 47.3% in 2011. In both years, approximately a quarter of men did not achieve any aerobic steps. BMI changed among participants from 25.6 to 26.1 during the follow-up period (*p* < 0.001) and WtHR from 0.51 to 0.53 (*p* = 0.001). Average weight gain between the study years was 2.6 kg among women and 1.1 kg among men (*p* < 0.001). BMI change had a significant correlation with step change (*r^2^* = −0.146, *p* = 0.001) and aerobic step change (−0.075, *p* = 0.005). WtHR change had a significant correlation with step change (*r^2^* = −0.100, *p* = 0.01) and aerobic step change (*r^2^* = −0.091, *p* = 0.05).

**Figure 1:**
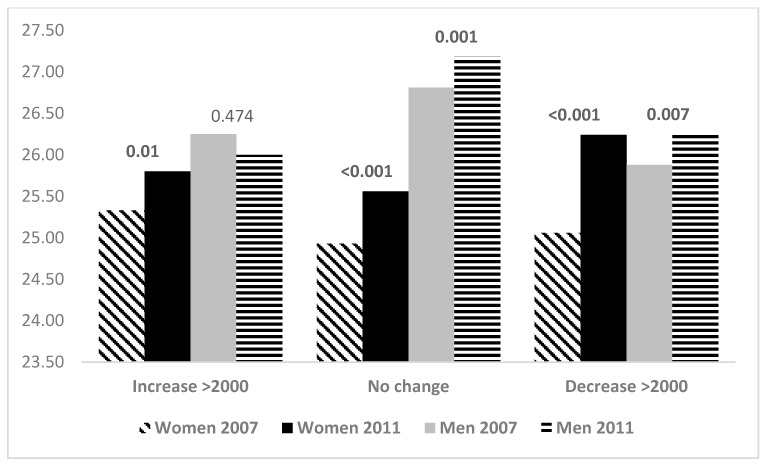
BMI in 2007 and 2011 in women and men according to change groups (increase, maintain or decrease) of total steps from 2007 to 2011. *p*-values for difference between 2007 and 2011.

**Figure 2:**
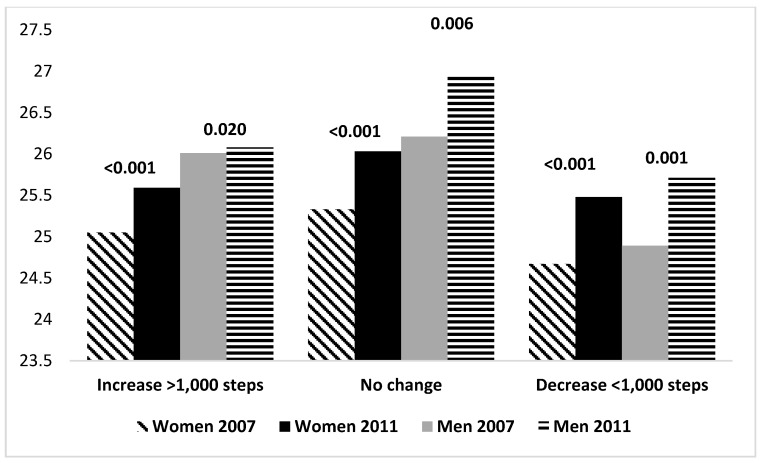
BMI change in the different aerobic step change groups among men and women with *p*-values, 2007–2011.

BMI levels in 2007 and 2011 are shown for increases’, maintainers’, and decreasers’ PA groups in Figure 1 and Figure 2. Among those who increased their daily steps by more than 2000, BMI stayed at the same level (*p* = 0.077). Among those who maintained their daily steps, BMI increased (*p* < 0.001). Among those, who decreased their daily steps BMI increased (*p* < 0.001). Among those who stayed inactive (*n* = 67) (<5000 steps in both years), BMI in 2007 (26.9) increased significantly to year 2011 (27.7) (*p* = 0.003). BMI increased in all aerobic step change groups. WtHR increased among women (*p* = 0.029–<0.001) both in total and aerobic step groups, while among men in increasers group WtHR did not change (*p* = 0.140–0.229).

## 4. Discussion

Unlike in earlier studies [9,10,11], in this study overall PA measured by daily steps seems to be increasing in midlife during four years follow-up period. Even though the change in total steps was not statistically significant, the direction is encouraging. These results support the previous results that BMI tend to increase during middle-age [9]. In this study, it was found that increasing daily steps was associated with no change in BMI, while decreasing steps or maintaining steps was associated with increases in BMI. This is in line with previous results that even though individuals increase their PA, weight may still increase [16].

These results are in line with previous studies suggesting that higher steps are associated with maintaining BMI in the same level [11]. Likewise, lower WtHR is associated with higher levels of PA [23]. Generally in high-income countries leisure-time PA has increased in adults, but at the same time, occupational PA has decreased [4]. In this study, aerobic steps had decreased which may indicate decreases in leisure time PA. The study population may be in a certain episode of life, when there is not so much time for leisure time PA, (e.g., they have family responsibilities). Percentages of inactive and low active participants in 2011 (47.3%) were a bit higher than in a Danish population (42.8%), but lower than in Brazilian adults (56.2%) [24]. Likewise, the percentage of those who were active (24.7%) in 2011 was a bit lower than in the Danish population 29.3%) [10], but clearly higher than in Brazilian adults (12.9%) [16]. The difference of a higher percentage of active Danish may be explained by a higher amount of active transport in Denmark. While 19.5% of Finnish people walk or cycle to work [25], in Denmark the percentage of those who solely cycle to work, is higher (25%) [26]. 

Even though the mean average of aerobic steps had decreased, the amount of those who had increased their aerobic steps was 51.5%. In addition, in Finland the proportion of those who were active (over 10,000 steps) increased from 19.0% (2007) to 24.8% (2011) while in Denmark among 18–75 years old adults, it decreased from 34.8% (2008) to 28.4% (2012). Likewise, in the Czech Republic, the amount of those who are defined as very active, are decreasing between 2008 and 2013 [27]. In Finland importance of PA has highlighted for years, and this may explain the reason why steps have increased during the follow-up period. It meay be, that in the Western countries same kind of phenomenom will turn up in recent years. 

In the future, more concern should be directed especially to those men, who are inactive, since they are more likely to maintain a sedentary lifestyle than women. Hence, more efforts should be directed towards low active men. Among women there were more changes in activity between the two occasions and steps increased. Recent studies have shown that among low active individuals, increasing PA from low levels can significantly influence a reduction in the mortality risk [28]. Among women more efforts should be directed to encouraging maintaining the PA. Even though there were plenty of women who increased PA, there were especially low active women at baseline who decreased their PA during the follow-up. On the other hand, more encouragement should be directed to all participant to maintain their physically active lifestyle and healthy eating habits.

To find out the stability of PA, a rigorous analysis should be done to determine the type of PA, what kind of sports keep people to be physically active and what kind of sports are more or less seasonal or occasional PA. Also further examination could be directed to life transitions, e.g., parenting, graduating, and moving to another city and how other health habits (e.g., diet) is related to these changes. 

Measuring PA with pedometers includes several limitations. For example, pedometers tend to under-detect certain activities such as cycling and are taken off during water-based activities, such as swimming [29]. Likewise, there is the possibility of recording of false steps during the day [30]. Hence, among some participants, there is the possibility of under-detection of PA. There are also several strengths in this study. Most of the objectively measured PA among adults rely on cross-sectional data, follow-up period is short, or study population is small. In this study, same population was followed four-year period and valid data was obtained from 1033 participants. In addition, usage of different anthropometric markers gives more reliability for the results. However, lack of diet data as a predictor of BMI and WtHR changes is a limitation in this study. In the Cardiovascular Risk Study, it has been found that some dietary habits (e.g., high intake of sweet beverages and high salt among women and high intake of French fries and milk) are related to weight gain [31]. In the following studies in the future, confounders, e.g., education, diet and socioeconomic status will be studied as a part of the study. In the future, step change and BMI and/or WtHR change studies should include data from eating habits and changes in other health behavior as well.

## 5. Conclusions

Increasing daily steps during middle age is associated with maintaining BMI at the same level. In this study, it was found that increasing daily steps to 2000 steps was associated maintain BMI at the same level as four years before. Since individuals’ metabolic rate declines with age, PA should be increased with age to maintain BMI at the same level as previously [32]. Maintaining PA at the same level as four years before was associated with an increase in BMI. Also, one of the issues emerging from this study is high stability of PA among men. Those who had low activity in 2007 were likely had low activity in 2011.

## Figures and Tables

**Table 1:** Changes in daily steps and aerobic steps from 2007 to 2011, *N* = 1033.

Change in Steps	% of Participants in Each Category		
**Total Steps/Day**	**All**	**Women**	**Men**
Increase ≥ 2000	25.3 (261)	24.8 (130)	25.8 (131)
Maintain	56.1 (579)	59.2 (311)	52.8 (268)
Decrease > 2000	18.7 (193)	16.0 (84)	21.5 (109)
Aerobic Steps */Day			
Increase (+1000)	24.7 (255)	25.1 (132)	24.2 (123)
Maintain	47.9 (495)	44.8 (235)	51.2 (260)
Decrease (−1000)	27.4 (283)	30.1 (158)	24.6 (125)

* Aerobic steps are those taken during activities that last for at least ten minutes without interruption at a pace of 60+ steps per minute.

**Table 2:** Baseline characteristics of the study participants in 2007 and 2011 (*N* = 1033).

Demographics	Women							Men						
	2007 (*n* = 641)			2011 (*n* = 641)			*p*-Value	2007 (*n* = 392)			2011 (*n* = 392)			*p*-Value
Age (years), mean (SD)	38.2 (5.0)			42.2 (4.9)				38.1 (5.0)			42.0 (5.0)			
Education, years mean (SD)	15.8 (3.3)			15.7 (3.4)				15.0 (3.4)			15.0 (3.5)			
Weight (kg)	69.9 (14.7)			72.5 (16.3)			<0.001	86.4 (15.5)			87.5 (16.0)			<0.001
Height (cm)	165.9 (6.0)			165.9 (5.7)				179.6 (6.6)			179.8 (6.6)			
BMI (kg/m^2^) mean (SD)	25.1 (4.6)			25.8 (5.0)			<0.001	26.5 (4.0)			26.7 (4.1)			0.008
WtHR	0.50 (0.07)			0.53 (0.08)				0.53 (0.06)			0.54 (0.07)			<0.001
Pedometer values (steps/day)														
Total steps/day	25th	Median	75th	25th	Median	75th		25th	Median	75th	25th	Median	75th	
Aerobic steps/day	6045	7702	9568	6239	8029	10,312		5202	7016	9093	5458	7049	9397	
	666	2092	3478	498	1820	3687		0	821	2280	0	759	1940	

## Abbreviations

BMI	Body mass index
YFS	Young Finns Study
HEPA	Health enhancing physical activity
PA	Physical activity
SD	Standard deviation
WtHR	Waist-to-height ratio
